# A Rare Case of Table Fork Ingestion Requiring Laparotomy

**DOI:** 10.7759/cureus.83138

**Published:** 2025-04-28

**Authors:** Akay Edizsoy, Serhan Akalin, Zeynep Simay Ergin, Hatice Barak, Salih Cokpinar

**Affiliations:** 1 General Surgery/Surgical Oncology, Adnan Menderes University Faculty of Medicine, Aydın, TUR; 2 General Surgery, Adnan Menderes University Faculty of Medicine, Aydın, TUR; 3 Thorcic Surgery, Adnan Menderes University Faculty of Medicine, Aydın, TUR

**Keywords:** endoscopic, fork, ingestion, laparotomy, table

## Abstract

Foreign body ingestion may occur accidentally or intentionally, particularly among children, elderly individuals, and those with psychiatric conditions. While small objects often pass spontaneously, large or sharp items may require endoscopic or surgical retrieval to prevent complications.

We present the case of a 22-year-old woman who accidentally ingested a table fork while playing with her children. Imaging revealed the fork’s prongs in the esophagus and the handle in the stomach. Endoscopic removal was attempted but aborted due to the size of the fork and the potential risk of esophageal injury. The patient subsequently underwent laparotomy, and the fork was retrieved via gastrostomy without complications.

Surgical removal should be considered when endoscopic retrieval of a foreign body is unsuccessful or carries a high risk, as illustrated in this rare case of table fork ingestion.

## Introduction

Foreign body ingestion can occur unknowingly during daily eating or drinking and may pass through the gastrointestinal tract without intervention. When detected during chewing, objects are typically expelled orally. However, some foreign bodies may be swallowed accidentally and cause clinical problems. These events are more common in children, individuals under the influence of alcohol or drugs, those with psychiatric disorders, and the elderly [1].

The prevalence of foreign body ingestion is particularly higher among pediatric and geriatric populations. Conditions such as dementia and Alzheimer’s disease are predisposing factors in the elderly, whereas attention-deficit disorders are key risks in children [2]. Non-toxic small objects may be managed conservatively, but larger or sharper items pose a significant risk of injury or perforation and often necessitate endoscopic or surgical extraction [3].

If not managed promptly, foreign bodies may cause injuries to the esophagus and surrounding organs. More distal complications in the GI tract may require complex surgical interventions. There are even reported cases of patients presenting with duodenal perforation due to accidental ingestion of a plastic fork [4].

## Case presentation

**Figure 1:**
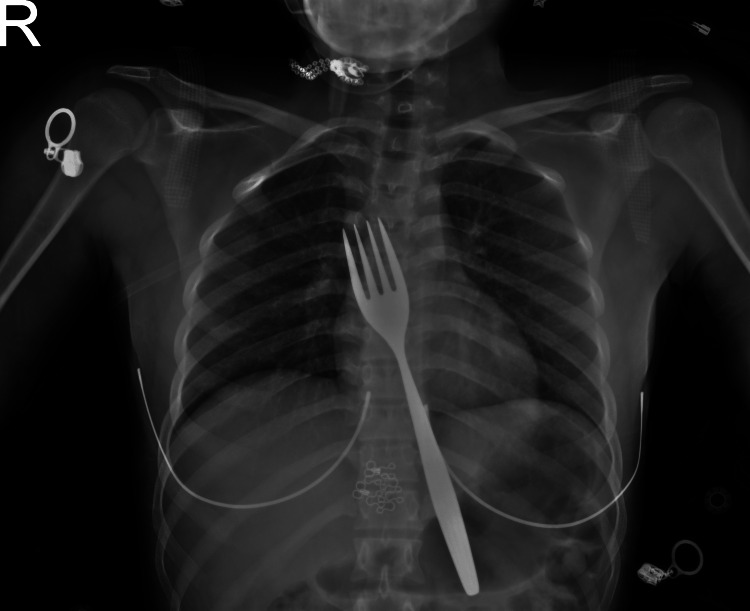
Chest X-ray showing the prongs of the table fork in the esophagus and the handle extending into the gastric lumen.

A 22-year-old Syrian female presented to Adnan Menderes University Emergency Department with complaints of epigastric pain and nausea. She reported accidentally swallowing a table fork while playing with her children. Her vital signs were stable, and she appeared calm. After the initial clinical evaluation, all laboratory tests, including serum Beta-human chorionic gonadotropin (HCG), were reviewed. At approximately the 30th minute following the patient’s arrival, a chest X-ray was performed, revealing the presence of a table fork. To assess for possible esophageal or adjacent organ injury, thoracic and abdominal computed tomography (CT) imaging was obtained at the 40th minute. No evidence of perforation or injury was identified. At the 60th minute, the patient underwent an upper endoscopy performed by the thoracic surgery team. Although no esophageal injury was observed, it was concluded that the foreign body could not be safely removed via endoscopy. Subsequently, the patient was transferred to the operating room at the 110th minute for surgical intervention. The duration of the laparotomy was 70 minutes. Chest X-ray imaging revealed the prongs of the fork in the esophagus and the handle extending into the gastric lumen (Figure 1).

**Figure 2:**
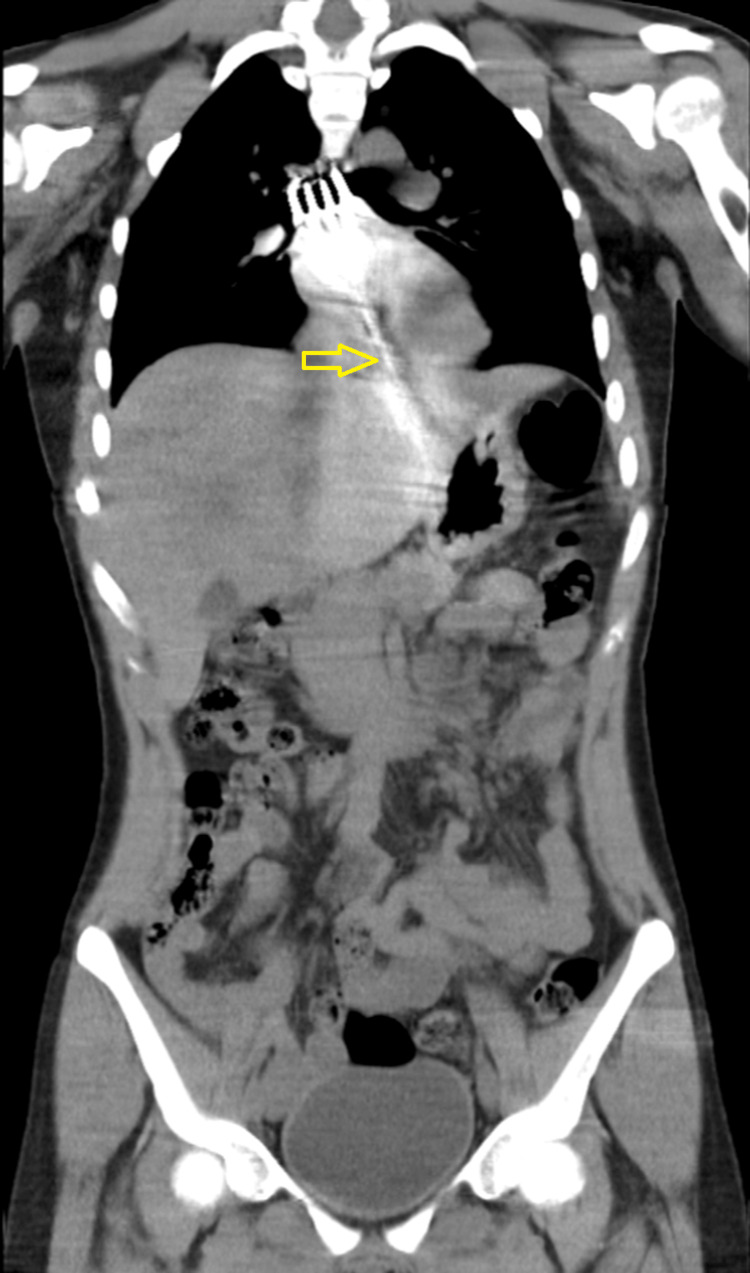
Abdominal CT confirming fork position without signs of perforation or hemorrhage. The gastroesophageal junction is marked by the yellow arrow.

Concerned about possible esophageal injury, thoracic and abdominal CT was performed, which confirmed the location of the foreign object and ruled out hemorrhage or perforation (Figure 2). Endoscopy was attempted by the Thoracic Surgery team, but the fork was too large to retrieve safely. Due to the risk of mucosal trauma, especially from the proximally positioned prongs, the procedure was aborted.

**Figure 3:**
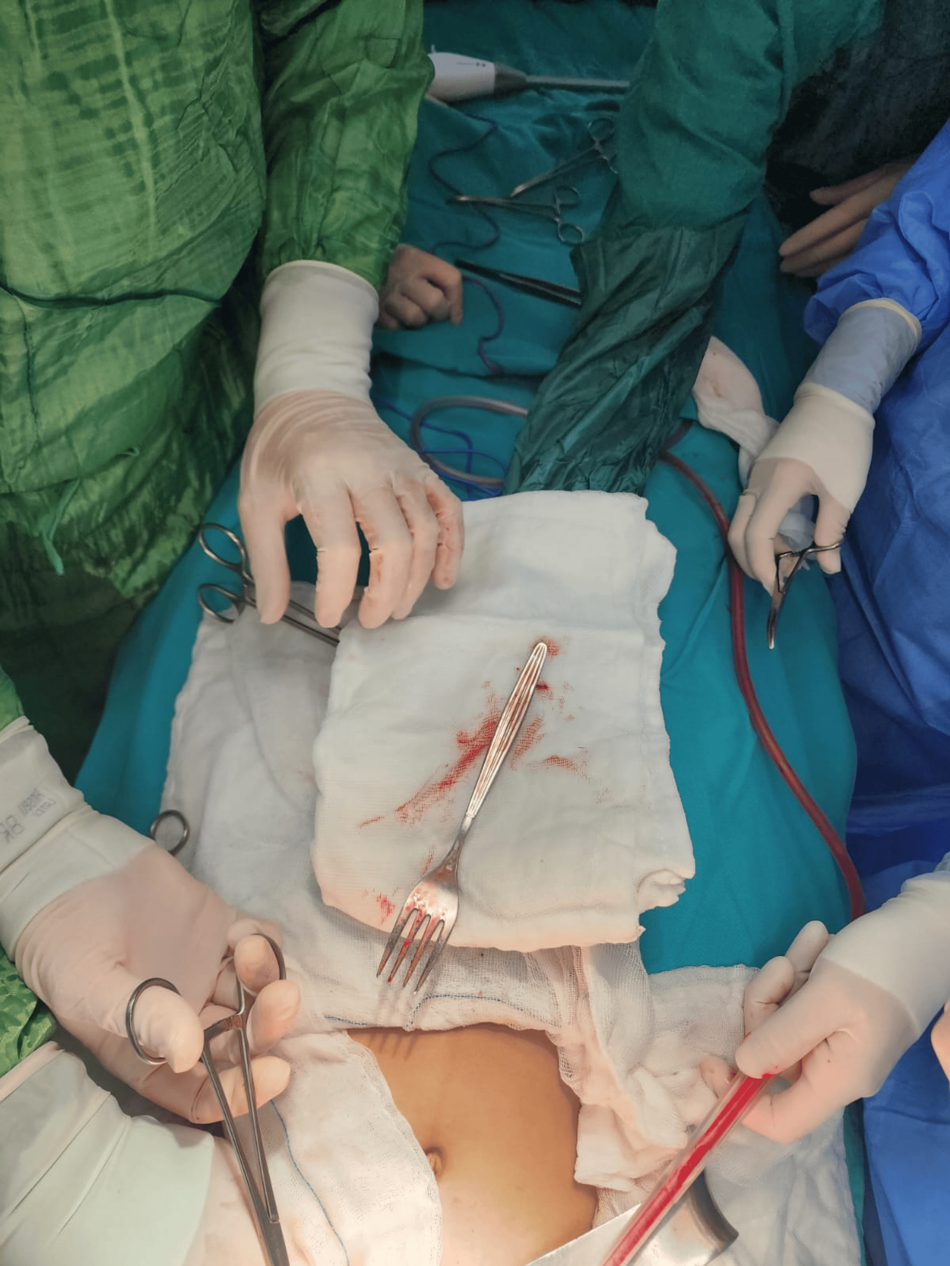
Intraoperative image during laparotomy with the fork being extracted via gastrostomy.

The patient was referred to General Surgery and taken to the operating room. A 4-cm midline epigastric incision was made, and laparotomy was performed. The serosa of the stomach was reached, and the fork handle was visible and palpable, pushing on the greater curvature. A gastrostomy was created at this site, revealing the handle of the table fork, which was gently grasped and extracted. The prongs were released through the gastroesophageal junction, and the fork was successfully removed from the abdominal cavity (Figure 3).

No intra- or postoperative complications were observed. Follow-up endoscopy showed no evidence of esophageal injury. The patient resumed oral intake and was discharged on postoperative day five. At outpatient follow-up, the patient had no complaints or complications.

## Discussion

According to the American Society for Gastrointestinal Endoscopy (ASGE) guidelines, for objects larger than 6 cm that have passed beyond the gastroesophageal junction, it is recommended that the object be grasped with a snare or basket and maneuvered into an overtube [5]. However, in our case, endoscopic removal was unsuccessful due to the rigidity and sharp edges of the fork. In a previous case, a broken plastic fork was successfully removed using endoscopic techniques and a double-scope method without esophageal injury [6]. However, in other reports, esophageal trauma during endoscopic retrieval led to the need for more complex surgeries and raised ethical concerns regarding the principle of "primum non nocere" [7]. In our case, the endoscopic procedure was appropriately discontinued to prevent potential esophageal damage. 

There is a case in the literature where ingested plastic forks perforated the ileum, leading to peritonitis. These patients were managed with laparoscopic surgery and primary repair [8]. In another case where endoscopic removal failed, laparotomy was performed as a definitive approach [9]. In a patient evaluated for the ingestion of a spoon and a knife, hydrothorax and pneumothorax were identified. Although the spoon was successfully removed endoscopically, a thoracotomy was required due to an esophageal injury caused by the knife [10].

Surgical intervention is required in approximately 1% of foreign body ingestion cases. Laparoscopy is considered a minimally invasive alternative for such interventions [11]. Although a laparoscopic approach could have been performed for our patient, we preferred laparotomy in this particular case to avoid missing a possible undetected injury and to prevent complications.

Cases of table fork ingestion are increasingly reported. While endoscopic retrieval should remain the initial approach, the risk of complications must be carefully evaluated. Surgical intervention via laparotomy or laparoscopy should be considered when safety cannot be assured through endoscopy.

## Conclusions

Although endoscopic removal is generally the preferred initial approach for ingested foreign bodies, surgical intervention should be promptly considered when anatomical positioning or object characteristics present a high risk for complications. This case highlights the need for individualized decision-making to ensure patient safety in uncommon but potentially hazardous situations. In such cases, a patient-specific treatment strategy should be developed. Both endoscopic and surgical teams should be readily available, and cases should be evaluated in collaboration with a multidisciplinary team.
